# Isolated Components From Spider Venom Targeting Human Glioblastoma Cells and Its Potential Combined Therapy With Rapamycin

**DOI:** 10.3389/fmolb.2022.752668

**Published:** 2022-03-14

**Authors:** Marcus Caballero, Natalia Barreto, Amanda Pires Bonfanti, Jaqueline Munhoz, Thomaz Rocha e Silva, Rafael Sutti, Liana Verinaud, Felipe Cezar Pinheiro de Mato, Guilherme Pauperio Lanfredi, Catarina Rapôso

**Affiliations:** ^1^ Faculdade de Ciências Farmacêuticas, Universidade Estadual de Campinas (UNICAMP), Campinas, Brazil; ^2^ Departamento de Biologia Estrutural e Funcional, Instituto de Biologia, UNICAMP, Campinas, Brazil; ^3^ Faculdade Israelita de Ciências da Saúde Albert Einstein, São Paulo, Brazil; ^4^ Faculdade de Ciências Médicas, Santa Casa de São Paulo, São Paulo, Brazil; ^5^ Departamento de Bioquímica e Imunologia, Faculdade de Medicina de Ribeirão Preto, Universidade de São Paulo (FMRP-USP), São Paulo, Brazil

**Keywords:** glioblastoma, animal venom, cancer therapy, cytotoxicity, mTOR, rapamycin

## Abstract

Glioblastomas (GBs) are responsible for a higher mortality rate among gliomas, corresponding to more than 50% of them and representing a challenge in terms of therapy and prognosis. Peptide-based antineoplastic therapy is a vast and promising field, and these molecules are one of the main classes present in spider venoms. Recently, our research group demonstrated the cytotoxic effects of *Phoneutria nigriventer* spider venom (PnV) in GBs. The present study aimed to select the purified PnV-components with potential antineoplastic effects, as well as to compare different metabolic conditions. Human GB (NG97) cells were treated with the PnV fractions: F1 (less than 3 kDa), F2 (between 3 and 10 kDa), and F3 (greater than 10 kDa). After treatments, viability (MTT), proliferation (CFSE), death (Annexin V/propidium iodide-PI), and cell cycle (PI) assays were performed. The F1 and F2 fractions in acute periods (1 and 5 h) and low concentrations (0.1 and 1 μg/ml) showed more relevant effects and were repurified in subfractions (SF1–SF11); from these, SF3 and SF4 showed the most significant effects. The previous inhibition of mTOR by rapamycin had a synergistic effect with SFs, reducing cell viability even more significantly than the untreated control. Taken together, the results point to components present in SF3 and SF4 as potential prototypes for the development of new drugs for GB treatment and stimulate studies to use these compounds in combination therapy with a rapamycin-like activity. Future studies will be conducted to characterize, synthesize the molecules, and to evaluate the efficacy and safety in preclinical models.

## Introduction

Gliomas are tumors originating from glial cells, mostly astrocytes (Castro et al., 2011), and represent 80% of all malignant primary tumors in the brain (Goodenberger and Jenkins, 2012). Gliomas classified as grade IV (glioblastoma, GB) are considered of high degree and greater malignancy (Weller et al., 2015). Since 2016, the classification of gliomas has not only been based on histopathological characteristics but also according to molecular parameters, such as mutation in isocitrate dehydrogenase (*IDH1* and *IDH2*) and codeletion 1p/19q (Louis et al., 2016). Gliomas also show changes in several other genes, such as *EGFR* (epidermal growth factor receptor), *P53*, *NF1* (neurofibromin 1), *CDKN2A/B* (cyclin-dependent 2A/B kinase inhibitor), and *PTEN* (phosphatase and tensin homolog, which inhibit the mTOR pathway). The overexpression and/or mutation of *EGFR* is often found in GBs, which subsequently leads to the activation of many downstream signal pathways such as the phosphatidylinositol 3-kinase (PI3K)/AKT/mTOR pathway (Li et al., 2016), and this pathway is one of the almost inevitably altered molecular pathways in *IDH*-wild-type GB (Le Rhun et al., 2019). However, the contribution of several of these mutations for targeted therapy development remains unclear (Giering et al., 2017).

GB is the most aggressive of malignant brain tumors (Bleeker et al., 2013), and also one of the most lethal, with an expected survival of 12–15 months after diagnosis; only 5% of patients survive more than 5 years (Gallego, 2015). The treatment of GB is based mainly on surgical resection, which can be associated with radiotherapy and chemotherapy with temozolomide (Lim et al., 2018). Given such a poor prognosis, new therapeutic approaches are needed.

Natural products have been used as a source for screening and developing new drugs. Currently, more than 50% of the drugs used in the world are derived from natural products (Choene and Motadi, 2016; Lin et al., 2020). It has been shown that biomolecules from scorpion and spider venoms have a chemotherapeutic effect on glioma, neuroblastoma, leukemia, lymphoma, breast cancer, lung cancer, hepatoma, and pancreatic and prostate cancer, among others (Rapôso, 2017). Our group demonstrated that *Phoneutria nigriventer* spider venom (PnV) decreased cell viability and proliferation, impaired cell cycle and induced apoptosis of human GB lineages (Barreto dos Santos et al., 2019), and reduced or eradicated the development of GB in a preclinical trial developed in mice (Bonfanti et al., 2020), and PnV-isolated components impaired cell migration and adhesion through RhoA-ROCK and Na^+^/K^+^-ATPase (Barreto et al., 2020).

Continuing the investigation, the objective of this study was to select fraction(s) and subfraction(s) from the PnV, which have the most significant effect on GB cells, considering survival, proliferation, and cell cycle. Additionally, we investigated these effects with mTOR inhibition by rapamycin, simulating the impairment of this pathway, commonly deregulated in this type of tumor (Mecca et al., 2018).

## Materials and Methods

### *Phoneutria nigriventer* Venom Production and Fractionation

Two samples of lyophilized crude venom were extracted by electrical stimulation of numerous adult spiders of both sexes (Sisgen A23162A). The crude PnV, fractions, and subfractions were stored at −80°C and dissolved in a sterile culture medium immediately prior to use. The composition and reproducibility of the venom lots were verified by high-performance liquid chromatography (HPLC). The initial fractionation of the crude venom was performed using the Amicon ultra centrifugal filter (#UFC801008; Thermo Fisher Scientific, Suwannee, GA, United States). This procedure consisted of the separation of the crude venom by molecular mass using molecular filters, generating 3 main fractions that were denominated: F1 (LW = low weight, less than 3 kDa), F2 (IW = intermediate weight, between 3 kDa and 10 kDa), and F3 (HW = high weight, more than 10 kDa). From these, experiments were conducted to select the more significant fraction, considering the effects presented; then, F1 and F2 were chosen, and F3 was eliminated. A new purification of F1 and F2 together, using HPLC, was carried out, obtaining new components, which were denominated subfractions 1 to 11 (SF 1–SF 11). Reversed phase HPLC was performed using a Shimadzu VP-ODS column, with 0.1% trifluoroacetic acid (TFA) as the mobile phase and 90% acetonitrile 0.1% TFA as the eluent.

### Cell Culture Maintenance and mTOR Pathway Inhibition

Human GB (NG97) cells were donated by a patient from the Hospital das Clínicas/Universidade Estadual de Campinas (HC/UNICAMP), and the cell line was established and characterized in a sequence of published studies (Machado et al., 2005; Schenka et al., 2005; Machado et al., 2008; Machado et al., 2009; Lin et al., 2020). NG97 cells were seeded at a density of 1 × 10^4^ per cm^2^ in a 25 cm^2^ culture bottle and were grown using Iscove’s modified Dulbecco’s medium (IMDM) containing 10% fetal bovine serum (FBS) and 100 UI/ml penicillin and streptomycin (pH 7.4) (Gibco), referred to as complete IMDM. The cell culture was maintained in a humidified atmosphere at 37°C and 5% CO_2_ until semi-confluence (about 90% of the total surface area). For the assays, cells were transferred after careful scraping of 48- or 96-well plates (Corning, Inc.), at an initial density of 1 × 10^4^ cells per well, and incubated at 37°C for 72 h. The cells were then treated with 14 μg/ml and 280 μg/ml of PnV (Rapôso, 2017), F1, F2, and F3 (0.1, 1, and 10 μg/ml) (Barreto dos Santos et al., 2019), or SF 1–11 (0.1 and 1 μg/ml), for 1, 5, 24, and/or 72 h, depending on the method (Barreto dos Santos et al., 2019; Barreto et al., 2020), according to the following assays, while the control cells were maintained in the medium for the same times. For the mTOR inhibition assay, cells were incubated with 50 nM of rapamycin (#051357-13 Cayman Chemical) (Foster and Toschi, 2009). The inhibitor was first diluted in DMSO to a concentration of 10 mM, and then diluted with IMDM to the final concentration. GB NG97 cells were incubated for 1 h prior to the treatments. Subsequently, the inhibitor was replaced during the treatment periods (1, 5, 24, and/or 72 h) together with SF 1–11.

### Cell Viability Assay

Thiazolyl blue tetrazolium bromide (MTT), whose reduction capacity indicates cellular activity, was used to determine the cytotoxicity of PnV, and its fractions and subfractions. MTT was added to each well and incubated at 37°C for 4 h, according to the manufacturer’s protocol. Acidified isopropanol was added to each well to solubilize the blue crystals of MTT. Absorbance at 540 nm, which indicates cell activity, was determined using a Multiskan GO microplate spectrophotometer (Thermo Fisher Scientific, Inc., Waltham, MA, United States). The MTT assay was performed with PnV 14 and 280 μg/ml and fractions (F1, F2, and F3; 0.1, 1, and 10 μg/ml, respectively), at 1, 2, and 5 h treatment times; after choosing and repurifying F1 and F2, the test was performed again using all SF (1–11), with or without mTOR inhibition, as mentioned before.

### Cell Proliferation Assay

After culturing, 2 × 10^4^ cells/mL were washed with sterile PBS and then resuspended in 1 ml PBS containing the CSFE probe (carboxyfluorescein succinimidyl ester, 5 μM) and maintained at room temperature for 5 minutes, protected from light. Then, complete IMDM was added to block the effect of CFSE, and the suspensions were washed twice by centrifugation at 300 g for 10 min, according to the manufactures’ protocol. Cells were then resuspended with complete IMDM, cultured, and treated with F1, F2, and F3 (0.1, 1, and 10 μg/ml). The culture was kept at 37°C, CO_2_ 5%, for 24 and 72 h. The analysis of an aliquot of cells labeled with CSFE by flow cytometry was performed on the same day of labeling to define the maximum incorporation value of the probe. After treatment time, cells from each well were transferred to appropriate tubes and analyzed using the FACSVerse flow cytometer (BD Biosciences), and the results were computed using the BD FACSuite software.

### Apoptosis/Necrosis Assay and Cell Cycle Analysis

To determine the extent of apoptosis and necrosis, after treatments with F1, F2, and F3 (0.1 μg/ml) for 1, 5, and 24 h, respectively, 2 × 10^5^ cells were stained with fluorescein isothiocyanate (FITC)-conjugated Annexin V and propidium iodide (Pi), using the Annexin V-FITC Apoptosis Detection Kit (Biolegend, San Diego, CA, United States #640914), following the manufacturer’s instructions. A total of 10,000 cells were analyzed, and apoptosis/necrosis was determined using the FACSVerse cytometer and FACSuite system (BD Biosciences). The data were analyzed using FlowJo software (v7.6.5). For cell cycle distribution, following the treatments, 2 × 10^4^ cells were fixed overnight at 4°C in 70% ethanol. The fixed cells were then washed with PBS and incubated with 1 ml solution containing 50 μg/ml Pi, and 0.5 µg RNase A, for 30 min, in the dark. Cell cycle distribution was assessed by flow cytometry using the FACSVerse cytometer and FACSuite system (BD Biosciences, Franklin Lakes, NJ, United States), and the data were analyzed using FlowJo software (v7.6.5).

### Statistical Analysis

Values were analyzed using the GraphPad Prism software package, v. 5.0 (San Diego, CA, United States). The level of significance was analyzed using one-way analysis of variance (ANOVA) followed by Dunnett’s and Tukey’s multiple comparisons tests. Unpaired Student’s t-test was used to compare each treatment with the control. Error bars show the standard error of the mean (SEM). A *p*-value < 0.05 indicates the statistical significance.

## Results

### Venom Quality and Fractionation Scheme

**FIGURE 1:**
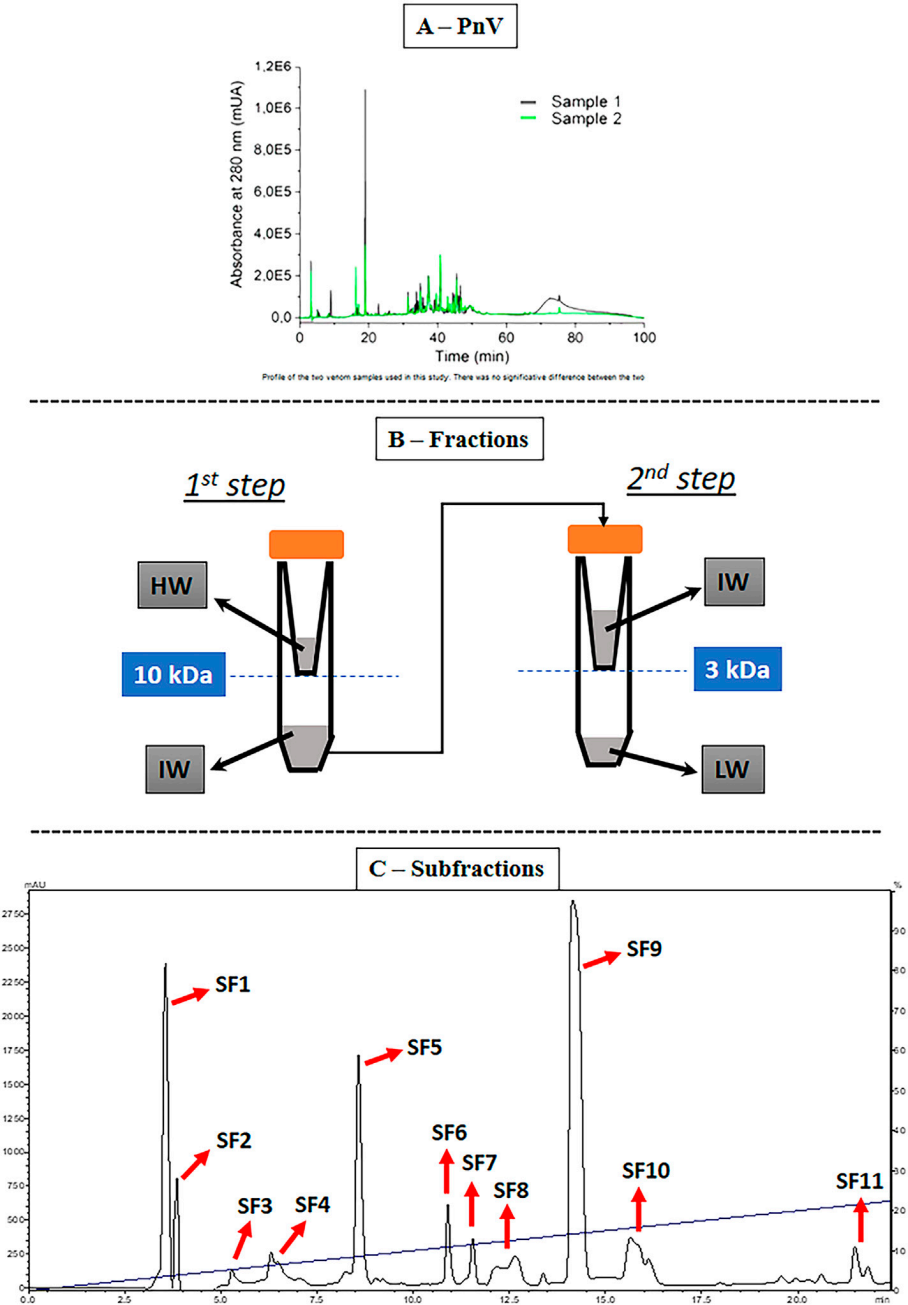
Venom samples and purification. **(A)** Venom profile by high performance liquid chromatography (HPLC). There was no significant difference between the two PnV samples used in this study. **(B)** Stages of the first PnV fractioning. This procedure consists in separating the fractions by molecular mass using molecular filters with nominal separation at 10 and 3 kDa. LW = low weight, less than 3 kDa (F1); IW = intermediate weight, molecules between 3 and 10 kDa (F2); HW = high weight, above 10 kDa (F3). **(C)** Fractions F1 + F2 together were repurified, obtaining 11 subfractions (SF1–SF11).

It was demonstrated there were no significant differences between the two extracted venom samples (Figure 1A). From the first PnV separation, three main fractions were obtained (Figure 1B). These fractions were still complex mixtures, and they were evaluated by the effects on viability, proliferation, death, and cell cycle to select those with the most significant effects on GB cells. Then, the purification of F1 and F2 (together) by HPLC generated eleven subfractions, named SF 1 to SF 11 (Figure 1C).

### Effectivity Reducing GB Cells’ Viability

**FIGURE 2:**
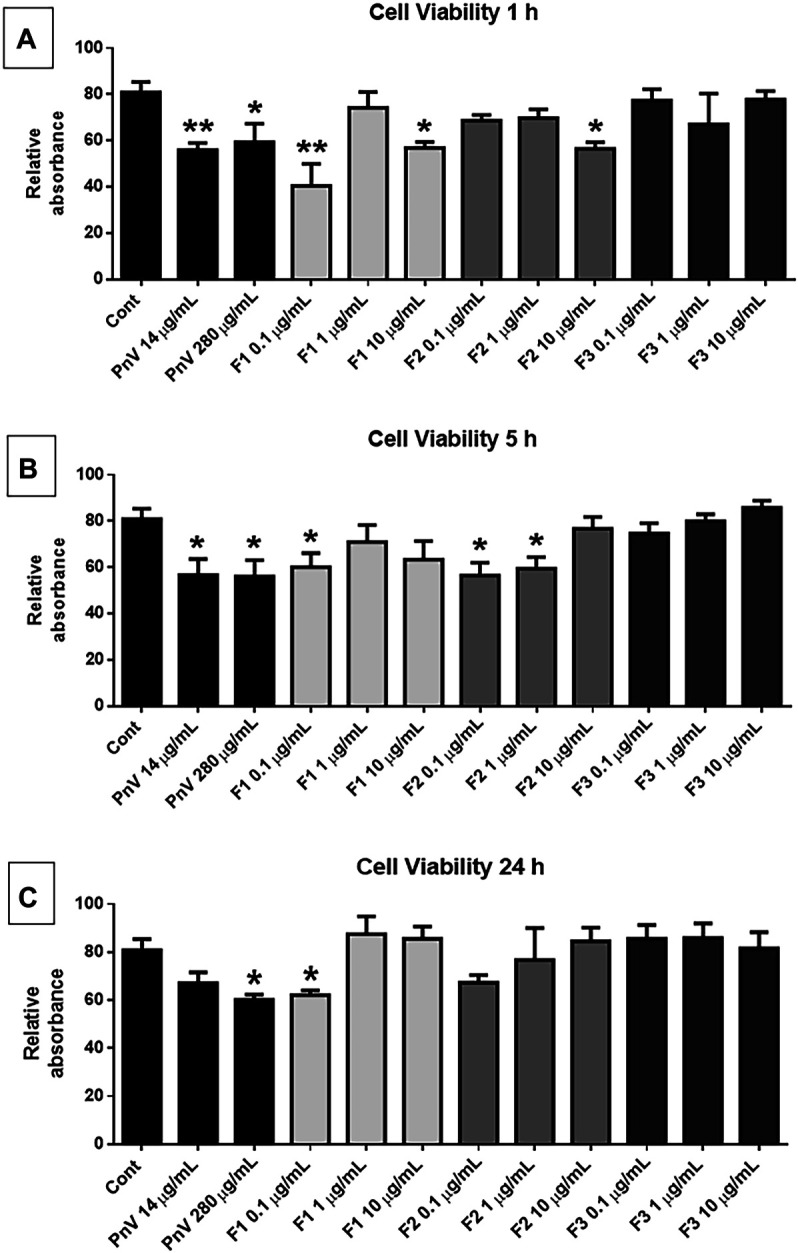
Cell viability assay (MTT): human GB cells treated with F1, F2, and F3 (0.1, 1, and 10 μg/ml). **(A)** After 1 h of treatment, F1 0.1 μg/ml was the most effective in reducing the viability of GB cells, compared to untreated control; F1 and F2 at 10 μg/ml were also significant. **(B)** After 5 h of treatment, F2 (0.1 μg/ml) significantly reduced the viability of GB cells. **(C)** After 24 h of exposure, no significant effect was observed. One-way ANOVA followed by Dunnett’s multiple comparisons test was performed; unpaired Student’s t-tests were used to compare each treatment with the control. *p < 0.05, **p < 0.01, ****p < 0.0001, compared to the control (Cont).

The cell viability assay (MTT) was performed using fractions F1, F2, and F3 (0.1, 1, and 10 μg/ml) (Figure 2). After 1 h of exposure, F1 at 0.1 μg/ml was the most effective treatment to reduce the viability of GB cells significantly compared to the untreated control (*p* < 0.01); F1 and F2, at 10 μg/ml, also induced a significant reduction in the viability of GB cells compared to the untreated control (*p* < 0.05), while no significant effect was observed in F3 fraction (2, A). After 5 h of exposure, the alterations were less significant than 1 h, and fractions F1 and F2 were still effective in reducing the viability of GB cells (*p* < 0.05) (2, B), while no significant effect was observed in F3 fraction. After 24 h of treatment, only F1 0.1 μg/ml showed a significant effect (2, C). Controls with PnV at 14 and 280 μg/ml induced a significant decrease of cell viability after 1 and 5 h, and after 24 h only the overconcentration (280 μg/ml) was significant.

### Cell Proliferation

**FIGURE 3:**
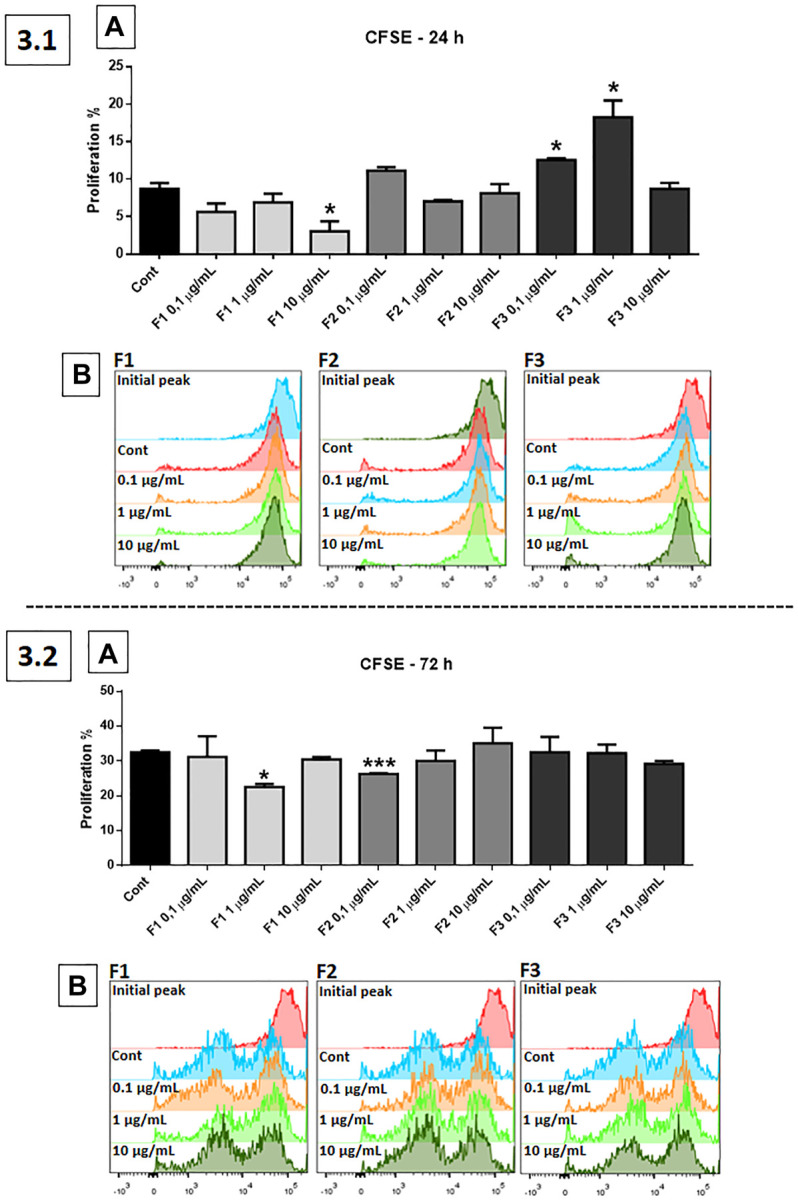
Proliferation test using CFSE probe: human GB cells treated with F1, F2, and F3 (0.1, 1, and 10 μg/ml). 3.1 **(A)** After 24 h of treatment, F1 at 10 μg/ml was the most effective in reducing proliferation, while F3 at 0.1 and 1 μg/ml significantly increased GB cell proliferation, compared to control. 3.1 **(B)** Representative histogram. 3.2 **(A)** After 72 h of treatment, F1 at 1 μg/ml and F2 at 0.1 μg/ml were significantly effective in reducing the proliferation of GB cells. 3.2 **(B)** Representative histogram. One-way ANOVA followed by Dunnett’s multiple comparisons test; unpaired Student’s t-tests were used to compare each treatment with the control. *p < 0.05, **p < 0.01, ***p < 0.001, ****p < 0.0001, compared to the control (Cont).

The cell proliferation assay (CFSE) was performed using fractions F1, F2, and F3 (0.1, 1, and 10 μg/ml) (Figure 3). In the 24 h of treatment, F1 at a dose of 10 μg/ml was significantly effective in reducing proliferation, compared to control (*p* < 0.01), while F3, at 0.1 and 1 μg/ml, was significantly effective in inducing GB cell proliferation (*p* < 0.05 and *p* < 0.0001, respectively) (3.1, A). Results are represented in the histogram (3.1, B). After 72 h of treatment, F1 at a concentration of 1 μg/ml (*p* < 0.05) and F2 at 0.1 μg/ml (*p* < 0.001) were significantly effective in reducing the proliferation of GB cells (3.2, A). Results are represented in the histogram (3.2, B).

### Induced Apoptosis and Necrosis in GB Cells

**FIGURE 4:**
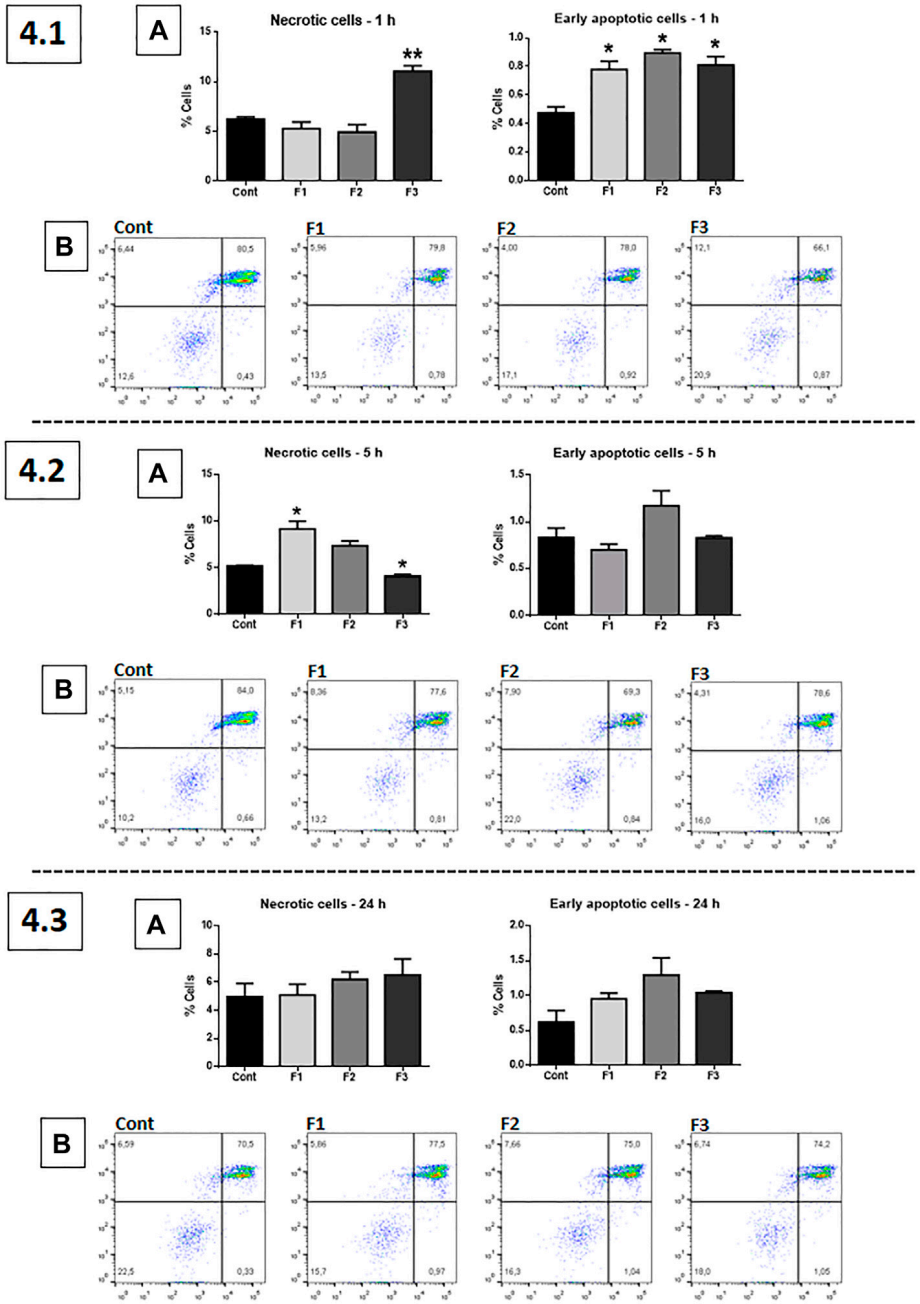
Apoptosis/necrosis assay (Annexin V/PI): human GB cells treated with F1, F2, and F3 at 0.1 μg/ml. 4.1 **(A)** After 1 h of treatment, F3 induced significant increase of GB cell necrosis, compared to the untreated control, whereas F1, F2, and F3 induced apoptosis in this time point. 4.2 **(A)** After 5 h of treatment, F1 and F2 induced significant necrosis, while F2 appears to induce apoptosis, although not significantly. 4.3 **(A)** After 24 h of exposure, no significant differences were observed. **(B)** 4.1, 4.2, and 4.3 show representative histograms. One-way ANOVA followed by Dunnett’s multiple comparisons test; unpaired Student’s t-tests were used to compare each treatment with the control. *p < 0.05, **p < 0.01, compared to the control (Cont).

The apoptosis/necrosis assay (Annexin V/PI) was performed using fractions F1, F2, and F3 at 0.1 μg/ml dose since this concentration induced distinctive effects in viability and proliferation tests (Figure 4). After 1 h of treatment, F3 induced a significant increase of GB cells necrosis compared to the untreated control (*p* < 0.01), whereas all fractions, F1, F2, and F3, were significantly effective in inducing apoptosis (*p* < 0.05) (4.1, A). After 5 h of treatment, F1 induced a significant increase of necrosis, compared to control; F2 apparently increased both necrosis and apoptosis, but it was not significant (4.2, A). F3 significantly decreased necrosis at this time-point. At 24 h of treatment, no significant differences were observed between the untreated control and the treated GB cells (4.3, A). Results are also represented in the histograms (4.1, B; 4.2, B; and 4.3, B).

### Cell Cycle Arrest at G1 and S Phases

**FIGURE 5:**
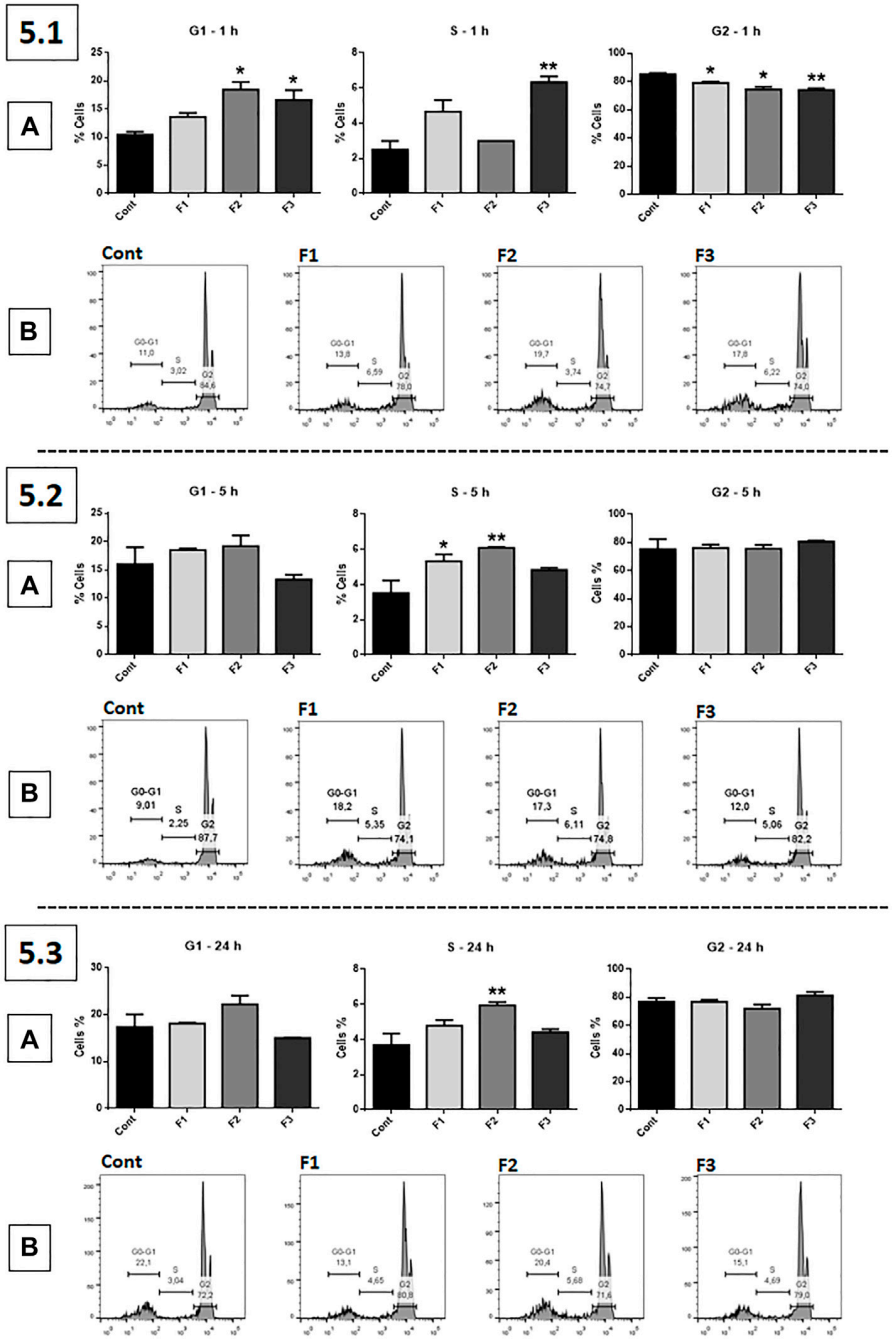
Cell cycle assessed by propidium iodide (PI): human GB cells treated with F1, F2, and F3 at 0.1 μg/ml 5.1 **(A)** After 1 h of treatment, F2 and F3 significantly increased the number of GB cells in the G1 phase of the cell cycle compared to the untreated control, while F3 accumulated cells in the S phase; all fractions significantly decreased the percentage of cells in the G2 phase. 5.2 **(A)** After 5 h of treatment, F1 and F2 significantly increased the number of cells in the S phase. 5.3 **(A)** After 24 h of treatment, only F2 significantly increased the number of cells in the S phase. **(B)** 5.1, 5.2 and 5.3 show representative histograms. One-way ANOVA followed by Dunnett’s multiple comparisons test; unpaired Student’s t-tests were used to compare each treatment with the control. *p < 0.05, **p < 0.01, compared to the control (Cont).

The cell cycle assay (Pi) was performed using fractions F1, F2, and F3 at 0.1 μg/ml (Figure 5). After 1 h of treatment, F2 and F3 significantly increased the percentage of GB cells in the G1 phase of the cell cycle compared to the untreated control, while F3 significantly increased cells in the S phase; F1, F2, and F3 significantly decreased the percentage of cells in the G2 phase (6.1, A). After 5 h of exposure, F1 and F2 significantly increased the number of GB cells in the S phase (5.2, A). At 24 h of treatment, only F2 significantly increased the number of GB cells in the S phase (5.3, A). The results are represented in the histogram (5.1, B; 5.2, B; and 5.3, B).

### Screening Subfractions’ Effects

**FIGURE 6:**
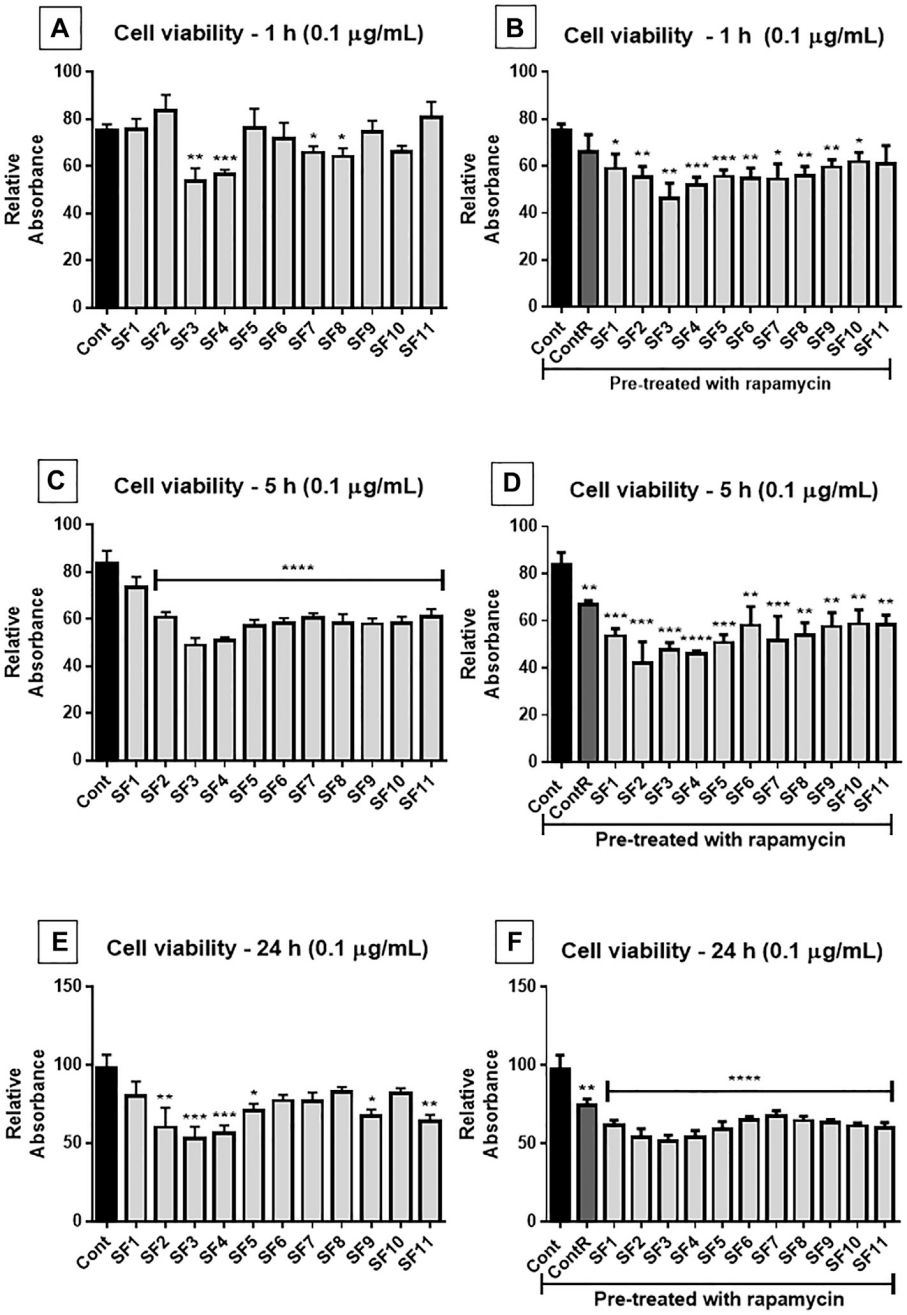
Cell viability assay (MTT): human GB cells treated with subfractions (SF1 to SF11) at 0.1 μg/ml, with or without mTOR inhibition with rapamycin. **(A)** After 1 h of exposure, SF3, SF4, SF7, and SF8 significantly reduced the viability of GB cells, compared to the untreated control. **(B)** Once the cells were pretreated with rapamycin, all SFs significantly reduced cell viability (except SF11). **(C)** 5 h after exposure, all SFs significantly reduced cell viability. **(D)** All SFs also reduced cell viability when they were pretreated with rapamycin. **(E)** After 24 h of treatment, SF2, SF3, SF4, SF5, SF9, and SF11 significantly reduced cell viability, and when pretreated with mTOR inhibitor, all SFs decreased viability of GB cells. **(F)** ANOVA followed by Dunnett’s multiple comparisons test; unpaired Student’s t-tests were used to compare each treatment with the control. *p < 0.05, **p < 0.01, ***p < 0.001, ****p < 0.0001, compared to the control (Cont).

The cell viability assay (MTT) was performed using subfractions SF 1 to SF 11 (0.1 and 1 μg/ml) from F1 and F2 separation by HPLC. The results showed, in general, that SF 3 and SF 4 were the most effective in decreasing viability of GB cells, and 0.1 μg/ml concentration was more effective than 1 μg/ml; when the cells were pretreated with rapamycin, the effect of SFs was exacerbated, except in 5 h of 0.1 ug/mL treatment (interestingly, the most effective time and concentration) (Figure 6).

This screening revealed that SF 3, SF 4, SF 7, and SF 8 subfractions at 0.1 μg/ml and 1 h of treatment (Figure 6A) were more effective in reducing the viability of GB cells than the control group. When cells were pretreated with rapamycin, SF 1 to SF 10 (Figure 6B) showed a reduction in cell viability compared to the control group, and the reduction was more significant than cells without the inhibitor. After 5 h of treatment (Figure 6C), all SFs showed a reduction in cell viability compared to the control group. In cells pretreated with rapamycin (Figure 6D), all SFs also showed a reduction of viability compared to the control group; however, the data were not as significant as those without mTOR inhibition. At the 24 h treatment (Figure 6E), SF 2, SF 3, SF 4, SF 5, SF 9, and SF 11 induced a significant reduction in cell viability compared to the control group. In the cells pretreated with rapamycin (Figure 6F) all SFs induced a decrease of viability compared to the control group, which was more statistically significant than that without the inhibitor.

**FIGURE 7:**
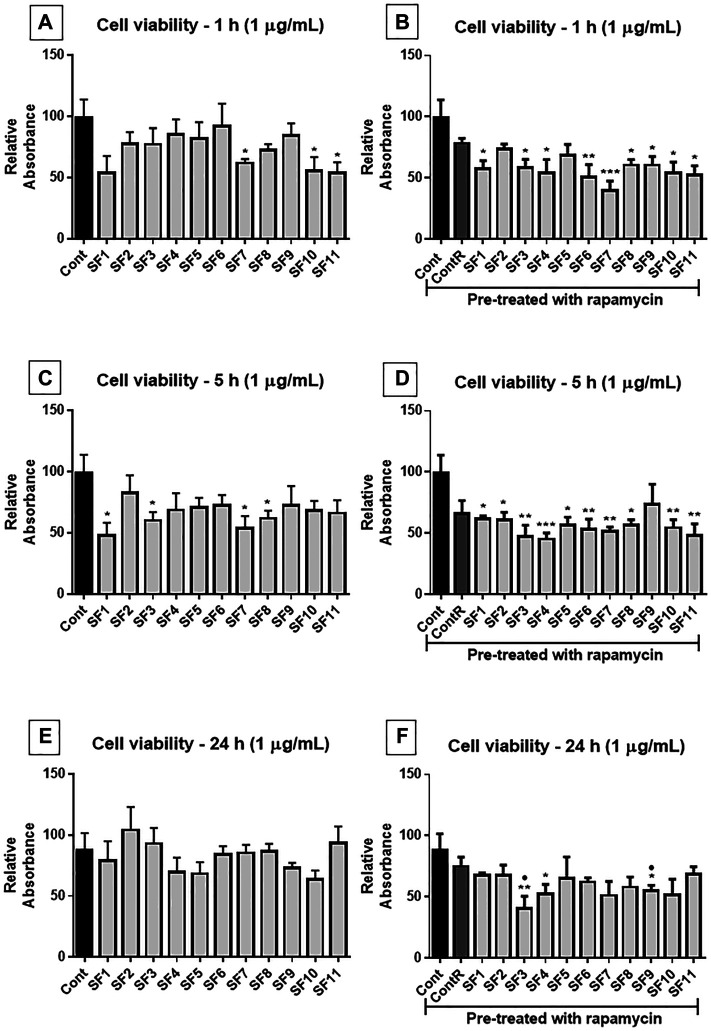
Cell viability assay (MTT): human GB cells treated with subfractions (SF1 to SF11) at 1 μg/ml, with or without mTOR inhibition with rapamycin. **(A)** After 1 h of exposure to treatments, SF7, SF10, and SF11 significantly reduced cell viability. **(B)** In cells pretreated with rapamycin, all SFs (except SF2 and SF5) significantly decreased the viability of cells. **(C)** 5 h after treatments, SF1, SF3, SF7, and SF8 significantly reduced cell viability, and when cells were pretreated with rapamycin **(D)**, all SFs except SF9 significantly reduced cell viability. **(E)** 24 h after exposure, no significant differences were observed, but when mTOR was inhibited **(F)**, SF3, SF4, and SF9 significantly reduced cell viability. One-way ANOVA followed by Dunnett’s multiple comparisons test; unpaired Student’s t-tests were used to compare each treatment with the control. *p < 0.05, **p < 0.01, compared to the control (Cont); *p < 0.05, compared to the control (Cont).

After 1 h of treatment, at 1 μg/ml (Figure 7A), the SFs 7, 10, and 11 showed a significant reduction of cell viability compared to the control group. In the cells pretreated with rapamycin (Figure 7B), all SFs, excepting SF 2 and 5, induced a significant reduction of viability compared to the control group. After 5 h of exposure (Figure 7C), SF 1, SF 3, SF 7, and SF 8 showed a reduction in cell viability compared to the control group. While cells were pretreated with rapamycin (Figure 7D), all SFs, except SF9, induced a significant reduction in cell viability compared to the untreated cells. At 24 h treatment (Figure 7E), no subfractions showed effective reduction in cell viability, and when the cells were pretreated with rapamycin (Figure 7F), SF 3, SF 4, and SF 9 showed a significant reduction in cell viability compared to the control group.

## Discussion

GBs correspond to more than 50% of gliomas and are characterized by their high mortality rate. As a heterogeneous and complex tumor, GBs represent a special challenge in terms of therapy and prognosis of cancer, so the development of new therapeutic approaches has great social relevance. Instead of focusing mainly on non-specific cytotoxic therapy, new substances targeting cancer-causing molecules can be a promising path in the treatment of GB. The spider venoms are potential sources of peptides with the ability to act on specific targets (Jenssen et al., 2006; Pineda et al., 2014). In the present study, the fractions and subfractions obtained from the PnV purification were tested in different concentrations (0.1, 1, and 10 μg/ml), to identify which of them have the most significant effect on inhibiting cell proliferation and survival of GB cells to drive to the drug prototype molecules.

It was verified that the fractions F1 and F2 in acute periods (1 and 5 h) were the most effective conditions to decrease tumor cells viability, inhibit proliferation, and induce cell death (mainly apoptosis) and cell cycle arrest. These results are in agreement with those of other studies showing spider venoms’ capability to stop tumor growth *in vitro*, to inhibit proliferation, and induce apoptosis or necrosis (Gao et al., 2007; Wu et al., 2019; revised by; Rapôso, 2017). Considering these results, F1 and F2 were purified together, resulting in the subfractions SF 1 to SF 11. From these, SF 3 and SF 4 at 0.1 μg/ml and 5 h of exposure were the best conditions to decrease GB cell viability. To better understand mTOR inhibition influence, rapamycin was used previously and during treatment with the SFs. The treatment with this inhibitor exacerbates the effects of SFs in almost all conditions, except at 5 h with 0.1 μg/ml, which interestingly is the condition that presented the best effect, suggesting a possible saturation of targets, which could limit the synergistic effect.

mTOR is a member of the serine/threonine protein kinase family that plays a central role in cell growth and proliferation (Duzgun et al., 2016). Also, mutations in the tumor suppressor gene *PTEN*, the protein that inactivates the PI3K/AKT/mTOR pathway, are frequent events in GBs and are associated with therapeutic resistance (Benitez et al., 2017). Therefore, the (PTEN)/PI3K/AKT/mTOR pathway has emerged as a crucial player in GB development and progression, and is a potential target for new drugs (Yang et al., 2019). However, it has turned out to be challenging to translate this extensive knowledge into a clinical benefit.

mTORC1 inhibitors mainly contain rapamycin (sirolimus) and its analogs, such as RAD001 (everolimus), CCL-779 (temsirolimus), AP23573 (ridaforolimus) (Li et al., 2016), and ABI-009 (nab-Sirolimus—an injectable nanoparticle form of human albumin-bound sirolimus) (Hou et al., 2019). Rapamycin inactivates mTORC1 by changing the kinase conformation. Although rapamycin and its analogs exhibit efficacy in non-clinical models (Arcella et al., 2013), trials of phases I and II have shown that these mTOR inhibitors are not as useful as single agents in GBs and would create hyperactivation of Akt and mTORC2. This could be caused by some feedback loop and pathway cross talk, where Akt can phosphorylate mSin1, leading to hyperactivation and promoting mTORC2 activation (Saxton and Sabatini, 2017), developing escape pathways (Chang et al., 2005; Cloughesy et al., 2008; Trials: NCT00515086—https://clinicaltrials.gov/ct2/show/NCT00515086—tested everolimus and was finished in 2011, NCT00022724—https://www.clinicaltrials.gov/ct2/show/NCT00022724—tested temsirolimus and was finished in 2018, and NCT00087451—https://clinicaltrials.gov/ct2/show/NCT00087451—tested ridaforolimus and was finished in 2015).

Other studies are exploring the combination treatment of rapamycin analogs with other modalities: trial NCT0062243 tested the combination of EGFR inhibitor erlotinib with sirolimus; however, it did not show promising results (Reardon et al., 2010); trial NCT00805961 (https://clinicaltrials.gov/ct2/show/NCT00805961), a phase II study of everolimus with bevacizumab (monoclonal antibody against VEGF), was feasible and efficacious (Hainsworth et al., 2012); and trial NCT03463265 (https://clinicaltrials.gov/ct2/show/NCT03463265) (recruiting) combines ABI-009 with bevacizumab, TMZ, lomustine (an alkylating nitrosourea compound), or marizomib (a marine-derived natural product proteasome inhibitor). Then, the selected SFs tested in the present study can be candidates that are used as combining therapy with mTOR inhibitors, potentiating the effects and/or avoiding the resistance mechanisms. In addition, considering the synergistic effect observed between SFs and rapamycin, it is possible to speculate that SFs can have members of the mTOR pathway as targets. In agreement with this hypothesis, it was shown that the crude PnV decreased Akt in acute periods of envenoming in the murine model (Mesquita-Britto et al., 2020). This mechanism in GB cells has to be confirmed.

Taken together, the results suggest that molecules present in SF 3 and SF 4 can be drug prototypes to develop a new chemotherapy against GBs. The best condition to continue investigating was established. In addition, the antitumor effects of the SFs were tested, indicating the action of molecules through the mTOR pathway, which represents a great challenge in the resistance to therapy. The molecules from SFs have potential to be used as combination therapy with rapamycin-like activity. Further studies will characterize the molecules and test the associated therapy in a syngeneic murine tumor model.

## Data Availability

The raw data supporting the conclusion of this article will be made available by the authors, without undue reservation.
